# A newly designed radiation therapy protocol in combination with prednisolone as treatment for meningoencephalitis of unknown origin in dogs: a prospective pilot study introducing magnetic resonance spectroscopy as monitor tool

**DOI:** 10.1186/s13028-015-0093-3

**Published:** 2015-01-31

**Authors:** Katrin Beckmann, Inés Carrera, Frank Steffen, Lorenzo Golini, Patrick R Kircher, Uwe Schneider, Carla Rohrer Bley

**Affiliations:** Division of Neurology, Clinic for Small Animal Surgery, Vetsuisse Faculty, University of Zurich, Winterthurerstrasse 260, CH-8057 Zurich, Switzerland; Division of Diagnostic Imaging, Vetsuisse Faculty, University of Zurich, Winterthurerstrasse 260, CH-8057 Zurich, Switzerland; Graduate School for Cellular and Biomedical Sciences, University of Bern, Freiestrasse 1, CH-3012 Bern, Switzerland; Division of Radiation Oncology, Vetsuisse Faculty, University of Zurich, Winterthurerstrasse 260, CH-8057 Zurich, Switzerland; Institute for Radiotherapy, Radiotherapy Hirslanden AG, Witellikerstrasse 40, CH-8032 Zurich, Switzerland

**Keywords:** Meningoencephalitis, Granulomatous, Necrotizing, Leukoencephalitis, Radiation, MRI, H-1 MRS, Dog

## Abstract

**Background:**

A plethora of treatment options have been described for canine meningoencephalitis of unknown origin (MUO), yet a gold standard has not been established. The aim of this prospective pilot study was to document the effect of a newly designed 30 Gray (Gy) radiation therapy (RT) protocol plus corticosteroids as treatment for focal and multifocal MUO, to monitor clinical and imaging changes during the course of the disease with conventional magnetic resonance imaging (MRI) and proton MR Spectroscopy (H-1 MRS) and to detect the occurrence of radiation related side effects.

**Results:**

Six dogs (3 with focal and 3 with multifocal lesions) were included in the study. The RT protocol used consisted of 30 Gy in 10 fractions. The neurological status of all six dogs improved during RT, with 3 of 6 cases returning to a normal condition. One dog was euthanized early during follow-up (<3 weeks after end of RT). Three month follow up MRI was normal in one dog and improved in 3 dogs and H-1 MRS normalized in 4. In the dog without improvement of the MRI lesions, the N-acetyl aspartate continued to decrease, while choline and creatine concentrations remained stable during that time. This dog was euthanized 18 month after the end of RT due to relapse. One dog was lost to follow up 12 month after completion of RT. The other 3 dogs are still alive at the time of writing.

**Conclusions:**

RT with 30 Gy in 10 fractions can provide an additional option for anti-inflammatory treatment of focal and multifocal MUO. The protocol used for treatment monitoring was feasible while no side effects of RT could be observed during the follow up period. Moreover, H-1 MRS could represent a new and non-invasive tool to control the progression of the disease during the treatment course.

## Background

Meningoencephalitis of unknown origin (MUO) is a term used for ante-mortem presumptive diagnosis of non-infectious meningoencephalomyelitis lacking definitive histopathological confirmation [1-4]. MUO includes a heterogeneous group of diseases with a suspected aberrant immune response directed against the central nervous system such as granulomatous meningoencephalitis (GME) [5-8], necrotizing meningoencephalitis (NME) [9], and necrotizing leukoencephalitis (NLE) [10,11]. The aetiology of these diseases is still unknown; however, a multitude of causes, such as neoplastic, genetic, autoimmune, infectious and toxic causes have been hypothesised but have not yet been proven [12-16].

Diagnostic imaging, in particular magnetic resonance imaging (MRI), plays an important role in the diagnosis of meningoencephalitis and the radiological features of this disease have been extensively reported in veterinary medicine [2,17-20]. However, MRI does not provide a definitive diagnosis, which is only achieved by histopathology. Proton magnetic resonance spectroscopy (H-1 MRS) is a non-invasive technique, which allows the determination of metabolites in living tissue [21]. H-1 MRS detects several metabolites in the normal brain, such as N-acetyl aspartate (NAA), choline (Cho), and creatine (Cr). NAA is considered a neuronal marker, and it is present only in neurons, axons and dendrites. Cho is involved in membrane synthesis and degradation, and Cr is involved in energy metabolism. Under pathological conditions these metabolites may show abnormal concentration (either absence, reduced or increased concentration); and other metabolites, normally not present in a healthy brain (such as lipids or lactate) may be evident [22,23]. H-1 MRS has been used to distinguish neoplastic from inflammatory disease in human patients [24,25] and has proven useful in monitoring the evolution of inflammatory processes such as multiple sclerosis [26]. The use of H-1 MRS in the brain of healthy dogs has been recently published [27,28]. It has also been used in research dogs for tumour models [29,30]. However, the clinical use of H-1 MRS in dogs with meningoencephalitis has so far not been described.

Various treatment options have been described for MUO, however a gold standard has not yet been established. In a recent meta-analysis [3] including 457 dogs from 71 studies, the authors were unable to identify a preferred immunosuppressive regimen. Radiation therapy (RT) protocols with total dosage ranging from 40–49.5 Gy, divided in fractions of 2.4 to 4.0 Gy in combination with corticosteroids have been described [31,32], leading to longer mean survival time in dogs with focal forebrain signs compared to corticosteroid monotherapy [31]. However, dogs with focal signs were not preferentially selected in the study of Munana *et al.* [31], only one dog with multifocal clinical signs was included in the RT group and this dog was euthanized one day after completing the RT. Sission *et al.* described a possible early delayed radiation reaction in one dog that received a whole brain RT protocol with 49.5 Gy in 15 fractions [32].

The aim of this prospective pilot study was to document the effect of a newly designed 30 Gy RT protocol applied in 10 fractions plus immunosuppressive dosage of corticosteroids as treatment for focal and multifocal MUO to monitor the clinical and the imaging changes during the course of the disease using MRI as well as H-1 MRS and to detect the occurrence of radiation related side effects.

## Materials and methods

Dogs recruited for this study were diagnosed with MUO between January 2012 and June 2013 in the Neurology Service of the Small Animal Department, Vetsuisse-Faculty, University of Zurich, Switzerland. Inclusion criteria included: (1) evidence of focal or multifocal brain lesions during the neurological examination without signs of spinal cord lesions or lesions in the peripheral nervous system; (2) abnormal cerebrospinal fluid (CSF) (reference interval: < 5 whit blood cells (WBCs)/μL, total protein < 0.3 g/L); (3) negative tests for infectious diseases in the CSF (4) evidence of focal or multifocal intra-axial lesions in MRI, according to previously reported features [2,17-20]; (5) H-1 MRS of the brain; (6) follow-up MRI, H-1 MRS, and CSF-centesis following the completion of and 3 months after RT; (7) during the RT and follow up period, no treatment with any other immunosuppressive medication beside prednisolone. For all patients, owner’s informed consent was obtained for treatment and follow-up. The official Animal Welfare Officer of the university approved the study design.

### Diagnosis

All dogs underwent thorough general examination and neurological evaluation performed either by a board-certified neurologist or by a neurology resident. In order to quantify the neurological changes of the patients during treatment and follow-up period, a neurodisability score (NDS) as described by Smith *et al.* [33] (Table 1) was applied at the time of initial presentation. Serum biochemical analysis and complete blood cell count were performed in all dogs. CSF was collected from the cisterna cerebellomedullaris and nucleated cell count, cytological examination and total protein (TP) concentration were determined. Tests performed to rule out infectious diseases included the following: a) polymerase chain reaction (PCR) from CSF for *Toxoplasma gondii* and Canine distemper virus (Clinical Laboratory of the Vetsuisse Faculty University of Zurich), b) real time PCR from CSF for *Neospora caninum* (Laboklin GMBH und co KG Bad Kissingen, Germany), c) European tick born encephalitis serology from serum, and CSF ELISA (enzyme linked immunosorbent assay) (Alomed Randolfzell –Böhringen, Germany).

**Table 1:** **Neurodisability score as described by Smith** ***et al.*** [33]

**Cranial nerves**			**Mentation**		**Postural responses**	
PLR abnormal	unilateral	0.5	Coma	1	Ataxic; ambulatory	1
	bilateral	1	Obtunded	1	Ataxic; nonambulatory	2
Menace response abnormal	unilateral	0.5	Circling/head pressing	2	Unable to stand	3
	bilateral	1				
Strabismus		1	**Seizures**		**Paresis**	
Nystagmus		1	Isolated	1	Paretic; ambulatory	1
Head tilt		1	Clusters	2	Paretic; nonambulatory	2
Facial paralysis	unilateral	1	Status Epilepticus	3	Plegia	3
	bilateral	0.5				
Absent gag reflex		1				
Dropped jaw		2				
Megaoesophagus		2				

#### Diagnostic imaging

MRI of the brain was performed with a 3 T MRI scanner (Philips Ingenia scanner, Philips AG, 8027 Zurich, Switzerland). Conventional images included T1-weighted (dorsal, sagittal, transverse), T2-weighted (dorsal, sagittal, transvers), FLAIR (transverse) and T1-weighted (dorsal, sagittal, trasverse), after contrast medium (Gadodiamid (Omniscan®), 0.3 mmol/kg, GE Healthcare AG, 8152 Glattbrugg, Switzerland). H-1 MRS comprised single voxel localization technique, acquiring Point-Resolved Spectroscopy (PRESS) with long and/or short echo-time (144 and 35 respectively). The single voxel was applied in the center of the main lesion, avoiding contamination with CSF and bony structures. Whenever possible, another single voxel was performed in the presumed normal contralateral aspect of the brain, used for comparison. When this was not possible (because of a diffuse lesion, lesion with considerable mass effect to the contralateral side, or lesion in the midline), database (using the same spectroscopy technique) obtained in our institution from normal healthy dogs was used.

A board certified non-blinded radiologist analysed the following features from the MRI studies: (1) number of lesions (focal, multifocal), (2) location, (3) margination (ill-defined, well-defined), (4) signal intensity pattern in all sequences compared to normal gray matter (hyperintense, hypointense or isointense), (5) contrast enhancement (degree: mild (defined as faintly identifiable signal increased to surrounding tissue in T1W post-contrast images), moderate (defined as easily identifiable signal increased to surrounding tissue in T1W post-contrast images), severe (defined as pronounced signal increased to surrounding tissue in T1W post-contrast images); pattern: homogeneous, heterogeneous, rim enhancement), (6) presence or absence of perilesional oedema and its extent (mild (defined as faintly identifiable T2W and FLAIR hyperintense signal surrounding the lesions), moderate (defined easily identifiable T2W and FLAIR hyperintense signal surrounding the lesions), severe (defined as readily visible T2W and FLAIR hyperintense signal well beyond the margins of the lesion) (7) presence or absence of mass effect.

LCModel software (LCModel, version 6.3, S Provencher, Oakville, ON, Canada. Automatic quantitation of localized in vivo 1H spectra with LCModel. *NMR Biomed* 2001;14:260–264.) was used to analyse the MRS data obtained. The following metabolites were included in this study: NAA (NAA, the sum of N-acetyl aspartate and N-acetylaspartylglutamate), total choline (tCho, predominantly glycerophosphocholine and phosphocoline, and creatine (Cr, the sum of creatine and phosphocreatine). Lactate and lipids were noted if present. Metabolite ratios compared to creatine were used: NAA/Cr, Cho/Cr, Cho/NAA. The results in the abnormal and presumed normal area of the brain were compared.

### Treatment

#### Radiation therapy

Treatment planning was performed on the basis of a three-dimensional computertomography (CT). For treatment planning the Eclipse External Beam Planning system version 10.0 (Varian Oncology Systems, Palo Alto, USA) was used, applying the AAA-algorithm (version 10.0.28). For planning CT and daily treatment, patients were under general anaesthesia, positioned in sternal recumbency, and immobilized in an individually shaped vacuum cushion (BlueBag BodyFix, Elekta AB, Stockholm, Sweden) and a custom-made bite block. The clinical target volume (CTV) (whole brain or lesion) was delineated on the CT and extended by 2 mm to define the planning target volume (PTV). Organs at risk were segmented (eyes). The prescribed dose was 30 Gy, delivered over two weeks in 10 daily fractions of 3 Gy to the ICRU reference point. Radiation was given by a Clinac iX (Varian Medical Systems, Palo Alto, USA). All 3D conformal radiation therapy (3DCRT) plans were calculated with 6 MV photons and consisted of five fields. Treatment was performed isocentrically with bolus and wedges to insure dose homogeneity. For some patients intensity modulated treatment (IMRT) was planned and delivered. The treatment was administered as image guided radiotherapy (IGRT) with daily orthogonal kV-imaging or cone beam CT (CBCT). Prior to treatment, all IMRT plans were dosimetrically verified using an Octavius**®**-Phantom (PTW Freiburg, Germany). All dogs received steroids during RT. The dosage was tapered depending on clinical signs (Table 2).

**Table 2:** **Summary of prednisolone treatment, cerebrospinal fluid (CSF) nucleated cell count, CSF total protein and neurodisability score (initial presentation to 12 month follow up)**

				**Prednisolone treatment (mg/kg SID) per day and duration of treatment**			**CSF cell count (cells/ μl)**			**CSF total protein (g/dl)**			**Neurodisability score**						
**Case**	**Age (month)**	**Breed**	**Sex**	**Before RT** ^**1**^	**During RT**	**After RT**	**Inital presentation**	**End of RT**	**3 months after RT**	**Inital presentation**	**End of RT**	**3 months after RT**	**Inital presentation**	**Beginnig of RT**	**End of RT**	**3 months after RT**	**6 months after RT**	**9 months after RT**	**12 months after RT**
1	8	French Bulldog	fs	2.1 (12 d)	2.1 (2 d)	0.25 (7 d)	20			0.4			1	1	1	/	/	/	/
					1.5 (4 d)														
					0.5 (9 d)														
2	9	Lagotto Romagnolo	m	1.9 (22 d)	0.9 2(2 d)	0.13 (7 d)	16.3	3	3	0.4	0.3	0.28	2.5	1	0	0	0	0	0
					0.45 (5 d)	0.07 (7 d)													
					0.25 (7 d)														
3	36	Pug	mk	3.5 (21 d)	2.2 (2 d)	0.5 (14 d)	126	4.3	4.33	1	0.3	0.33	3	4	2	1	1	1	1
				2.2 (35 d)	1.1(14 d)	0.25 (21d)													
						0.13 (21d)													
						0.07 (21d)													
4	72	Weimaraner	fs	4 (3 d)	2 (2 d)	0.2 (14d)	4.7	0.2	0.17	0.4	0.1	0.13	4.5	6.5	1.5	0	0	0	0
					0.8 (5 d)	0.1 (14d)													
					0.4 (7 d)														
5	156	Mixed breed	m	3.7 (2 d)	2.4 (2 d)	0.5 (21d)	244	3	6	1.4	0.5	0.45	5.5	2.5	0	0	0	0	0
				2.4 (8 d)	0.9 (12 d)	0.25 (7d)													
						0.13 (7d)													
6	15	Chihuahua	fs	4 (2 d)	2 (2 d)	0.5 (28d)	77	2	2	0.5	0.16	0.3	5	1.5	0	0	0	1	0
				2 (12 d)	1 (12 d)	0.3 (21d)													
						0.15 (21d)													

^1^RT: radiation therapy.

### Follow-up examinations

#### Follow-up

Clinical examination and the NDS were applied at the time of initial presentation and repeated before starting RT, after the last radiation treatment and followed by examinations at 3, 6, 9 and 12 month after RT. All follow-up examinations were performed by one of the authors (KB). Follow-up MRI and H-1 MRS and CSF tap were performed after the last RT and 3 months thereafter. Long term survival from beginning of RT to time point of writing this study was documented.

## Results

Seven dogs were enrolled prospectively in the study. One was later excluded because post mortem examination was compatible with viral encephalitis; however immunohistochemistry failed to demonstrate presence of the endemic viral disease causing encephalitis (canine adenovirus, parvovirus, canine distemper virus, European tick-borne encephalitis virus) and of rabies virus. The remaining 6 dogs included a French bulldog, a Pug, a Lagotto Romagnolo, a Weimaraner, a mixed breed and a Chihuahua. Three were spayed female and 3 were males (1 castrated and 2 sexually intact). Age at initial presentation ranged from 9 months to 13 years (mean 4 years, median 2 years). General clinical examination was unremarkable in all 6 dogs.

### Before radiation therapy

The range of the NDS at initial presentation was variable and all dogs had received steroid treatment prior to RT (Table 2). One dog was treated additionally with cytosine arabinoside (50 mg/m^2^ q 12 h for 2 d), but the dogs neurological status worsened and it showed side effects due to steroid treatment (polyuria-polydipsia, panting, alopecia and muscle weakness). RT was then offered as an alternative treatment. Cytosine arabinoside was not repeated and instead RT was started 56 days after the initial presentation (50 days after cessation of cytosine arabinoside). Before performing the planning CT, while the dog was free of any other immunosuppressive drug apart from steroids, a new complete study (MRI, H1- MRS and CSF tap) was performed. Only the latter results were included in the present prospective study and were then compared to those performed during the planned follow up.

The first RT was applied 3 to 56 days after starting the medical treatment (mean 19.5 days, median 13 days). Medical treatment before the first radiation treatment resulted in an improved NDS in three dogs, a worsened NDS in two (including the dog previously treated with cytosine arabinoside) and an unchanged NDS in one. The detailed dosages of prednisolone, CSF examinations results, and NDS are summarized in Table 2. MRI findings revealed 3 dogs with focal and 3 with multifocal lesions (Table 3).

**Table 3:** **Summary of the MRI findings before treatment of 6 dogs with meningoencephalitis of unknown origin, divided into dogs with focal and multifocal lesions**

	**Multifocal lesions (n = 3)**	**Focal lesions (n = 3)**
**Location**	White and grey matter frontal, parietal and occipital lobe, with thalamic involvement (1)	Brain stem to ventro-medial thalamus (2)
	White matter occipital lobe and optic nerve (1)	Cerebellar hemisphere (1)
	Corpus nucleus caudatus and temporal lobes (1)	Parietal white matter (1)
**Margination**	Ill-defined (3)	Ill-defined (3)
**Signal intensity**	T2W and FLAIR hyperintense (3)	T2W and FLAIR hyperintense (3)
	T1W hypointense (3)	T1W hypointense (2)
		T1W isointense (1)
**Contrast enhancement**	Mild and heterogeneous (3)	Moderate heterogenous (2)
		Moderate homogeneous (1)
**Perilesional edema**	Mild (2)	Mild (1)
	Moderate (1)	Moderate (2)
**Mass effect**	Present (3)	Present (3)

**Figure 1:**
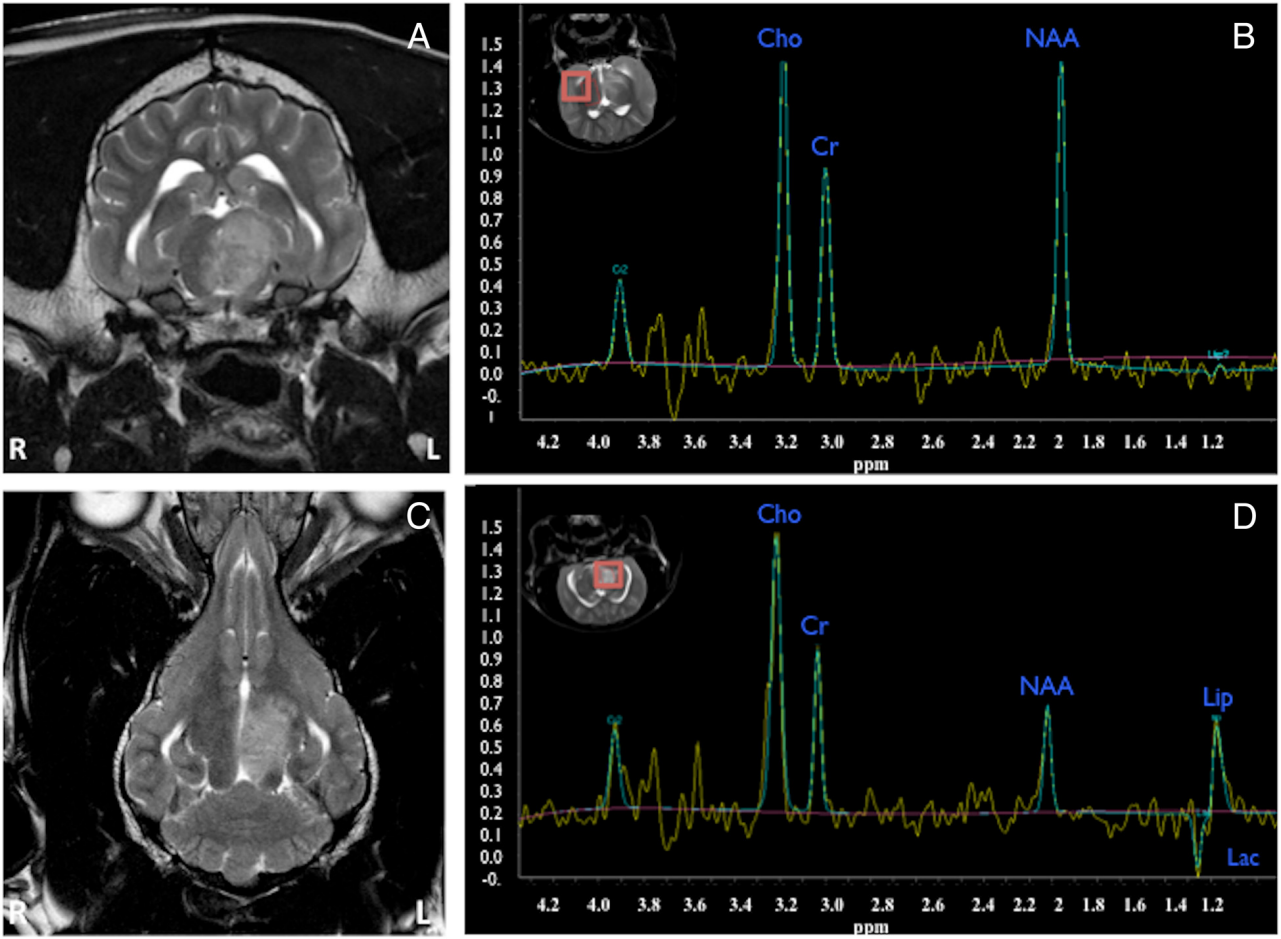
**Pre-treatment MRI (A and C, T2W sequences, transverse and dorsal respectively) and pre-treatment H-1 MRS (B and D, single voxel PRESS-144 on the normal and abnormal brain respectively; the red squares highlight where the voxel was located), of a Weimaraner dog with focal MUO affecting the left thalamus extending to the mesencephalon.** In **A** and **C** an ill-marginated hyperintense lesion is seen, causing mild mass effect. When comparing **B** and **D**, it can be appreciated a clear reduction of the NAA peak, mild increase of Cho and the presence of lactate and lipids in the affected side.

**Figure 2:**
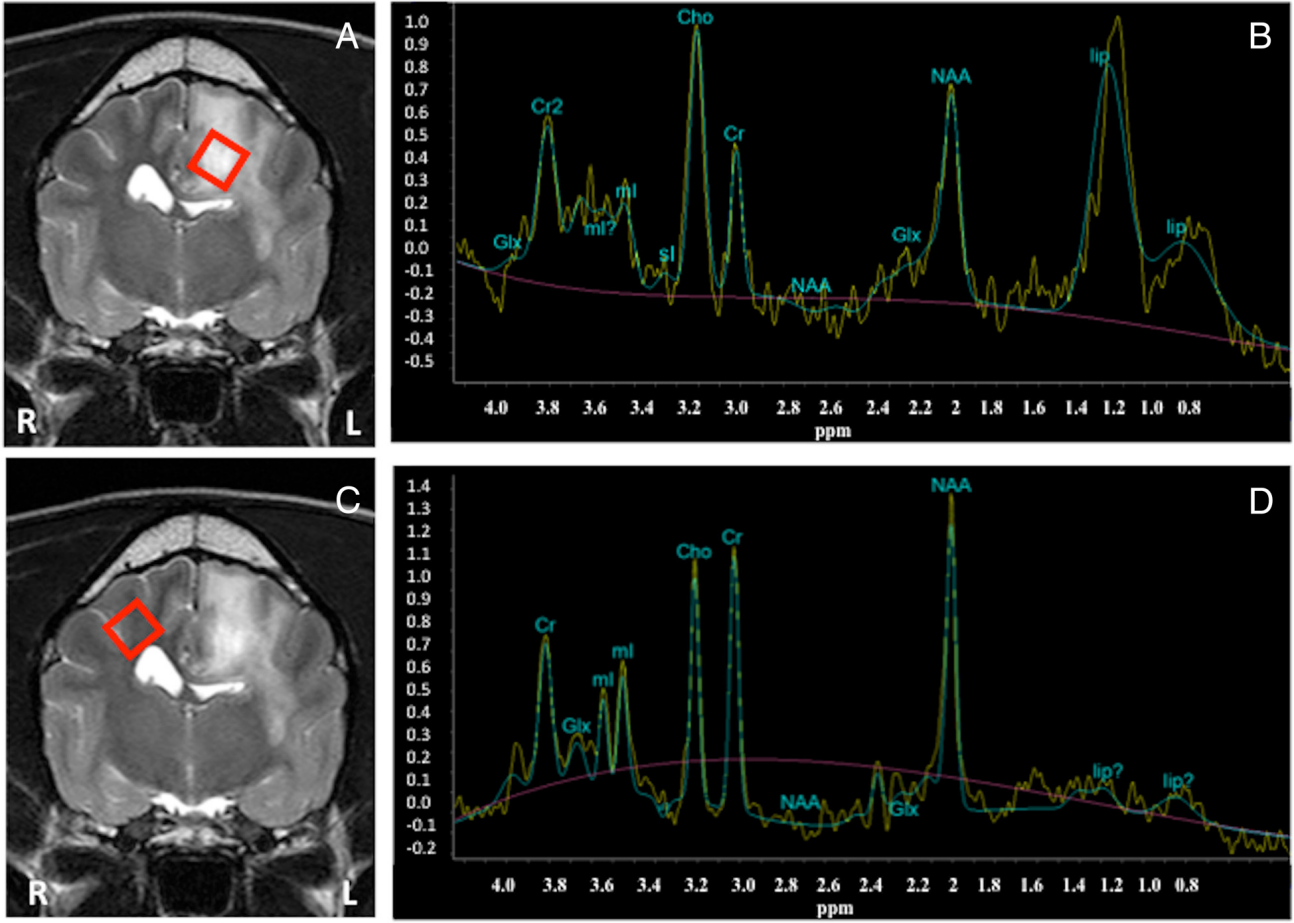
**Pre-treatment MRI (A and C, T2W transverse) and pre-treatment H-1 MRS (B and D, SV PRESS-35 on the abnormal and contralateral presumed normal area of the brain (the red squares highlight where the voxel was located) of a mixed breed dog with focal MUO affecting the left parietal lobe.** Notice in **A** and **C** the ill-defined hyperintense lesion, with moderate perilesional oedema and moderate mass effect compressing the left lateral ventricle. **B** shows mild elevation of the Cho peak, decrease of the NAA peak, and presence of lipids in the affected area.

**Figure 3:**
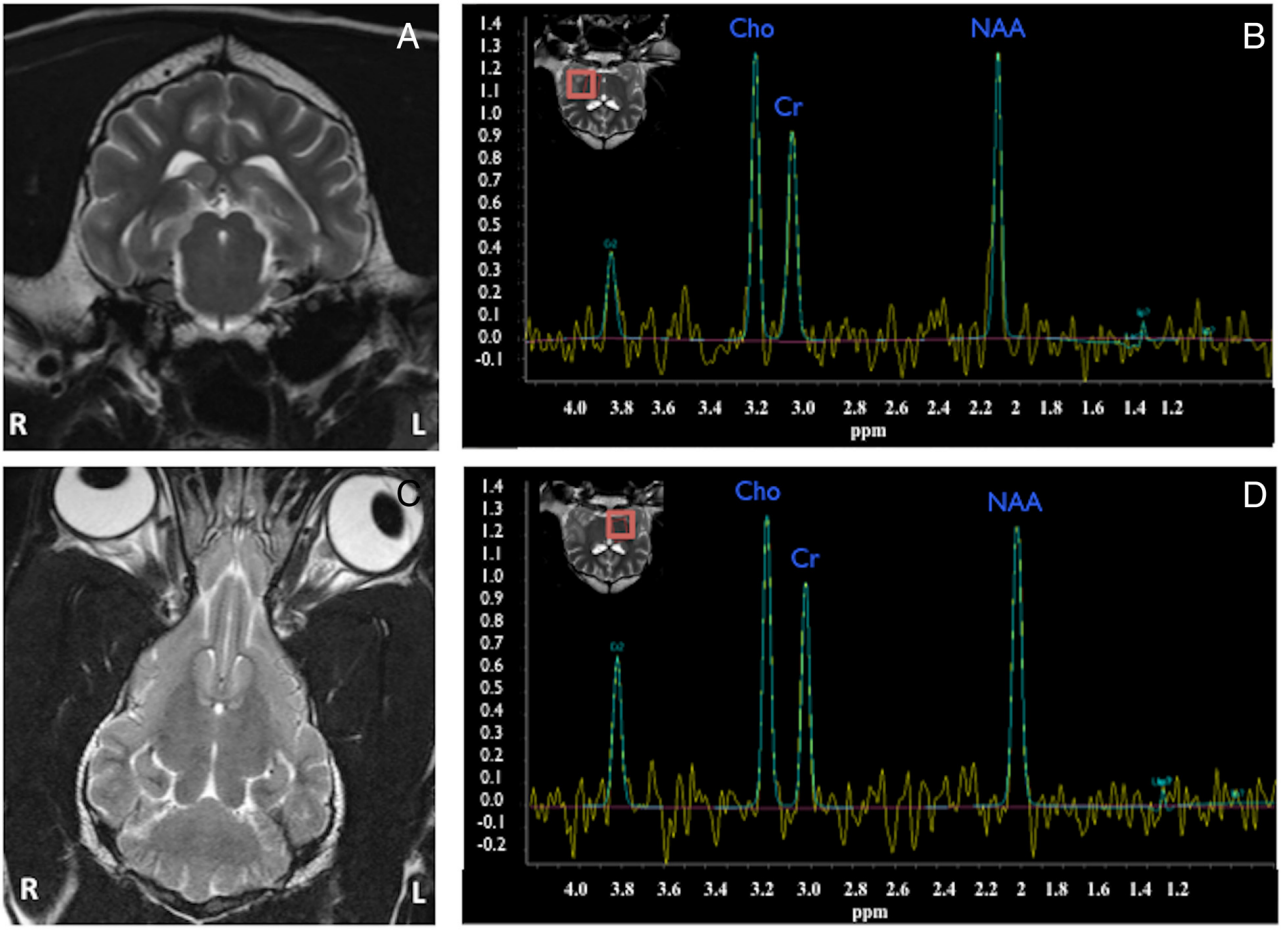
**The Weimaraner of Figure** **1** **just after radiotherapy treatment (A: transverse T2W, B: PRESS 144 normal side; C: dorsal T2W; D: PRESS 144 abnormal side).** The H-1 MRS single voxels were positioned at the same areas as pre-treatment. Notice the complete resolution of the lesion previously described affecting the left thalamus extending to the mesencephalon **(A** and **C)**. The spectrum of the normal **(B)** and previously abnormal region **(D)**, are now very comparable, revealing a recovery of the neurons.

H-1 MRS in all cases revealed low concentration of NAA when compared to the non-affected contralateral side of the brain (Figures 1, 2 and 3). The choline was either unchanged (2 cases, example Figure 1) or mildly increased (4 cases, example Figure 2). Lipids peaks were observed in 3 cases in the affected area (Figures 1 and 2), one of those also had a lactate peak (Figure 1). Lactate was detected in one additional case. The creatine peak tended to be rather similar in affected and non-affected sides, or very mildly reduced. All metabolite ratios before and after treatment are summarized in Table 4.

**Table 4:** **Summary of the H-1 MRS findings of 6 dogs with meningoencephalitis of unknown origin treated with radiotherapy, before, immediately after treatment and 3 month follow-up**

**Dogs**	**MRS before treatment**						**MRS after treatment**						**MRS follow-up after 3 months**					
	**NAA/Cr**		**Cho/Cr**		**Lips/Lac**		**NAA/Cr**		**NAA/Cho**		**Lips/Lac**		**NAA/Cr**		**Cho/Cr**		**Lips/Lac**	
	A	N	A	N	A	N	A	N	A	N	A	N	A	N	A	N	A	N
**1**	0.9	1.2	0.3	0.26	-	-	1.1	1.2	0.28	0.26	-	-	-	-	-	-	-	-
**2**	0.65	1.2	0.35	0.312	-	-	0.80	1.2	0.30	0.32	-	-	1.1	1.2	0.33	0.34	-	-
**3**	0.52	1.31	0.56	0.34	Lac	-	0.41	1.29	0.59	0.34	-	-	0.23	1.3	0.58	0.33	-	-
**4**	0.44	1.2	0.37	0.386	Lips	-	1.2	1.15	0.36	0.37	-	-	1.2	1.2	0.34	0.34	-	-
					Lac													
**5**	0.52	1.12	0.6	0.32	Lips	-	0.83	1.2	0.45	0.3	-	-	1.1	1.2	0.4	0.33	-	-
**6**	0.45	1.09	0.49	0.33	-	-	0.67	1.0	0.40	0.35	-	-	0.9	1.0	0.32	0.34	-	-

CSF pleocytosis was present in 5 out of 6 dogs (range: 5–134 WBCs/μL reference interval: < 5 WBCs/μL) and protein concentration was elevated in all dogs (range: 0.37 -1.41 g/L reference interval: < 0.3 g/L).

#### Radiation therapy

In all but one dog, the whole brain was considered as CTV (median volume 73.2 cm^3^, mean 59.8 cm^3^, range 1.1-80.6 cm^3^). The median dose to the PTV was 30.4 Gy (mean 30.4 Gy, range 27.6-31.4 Gy). Two patients were treated with an IMRT plan, as dose to organs at risk (eyes) was lower, while 3 patients had a regular 3D conformal RT (3DCRT)-plan. The median dose to the organs at risk (left and right eye) was 10.4 and 10.8 Gy (mean 10.5 Gy, range 0.1-16.5 and 10.8 Gy, range 0.1-16.9 Gy, respectively). Overall treatment time was 14 days (mean; range 12–16 days) and time to start of treatment after diagnosis was 17 days (mean; range 3 – 56 days).

### Follow-up immediate after treatment

The NDS was normal in 3 dogs (2 dogs with focal lesion, one with multifocal), improved in 2 dogs (one with focal, one with multifocal lesion) and unchanged in one dog (multifocal lesion) compared to the initial presentation and also before start of RT.

**Figure 4:**
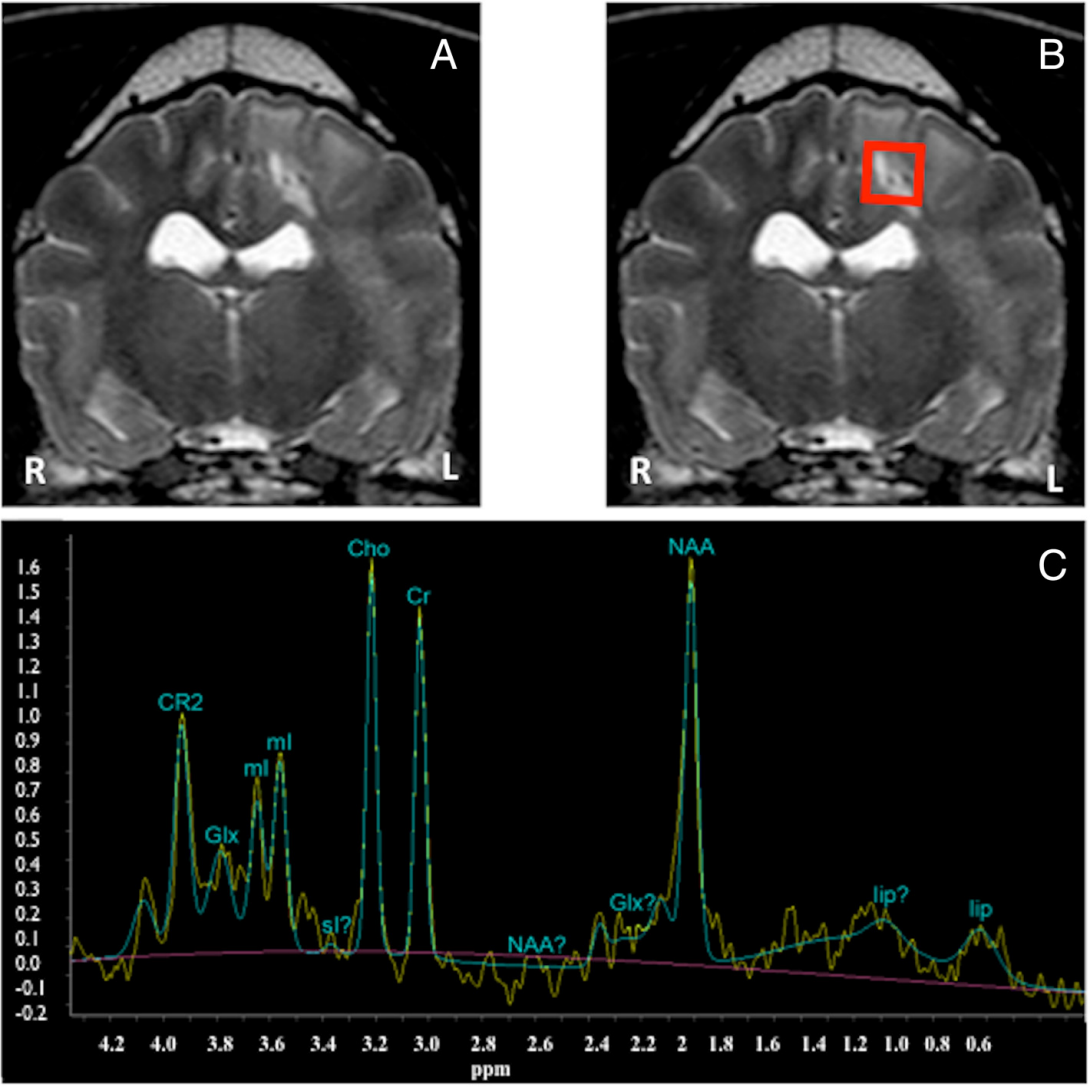
**The mixed breed of Figure** **2** **after radiotherapy treatment (A and B: transverse T2W, C: H-1 MRS single voxel PRESS 35 of the left temporal lesion).** Notice in A and B a reduction in size of the lesion, as well as improvement of the perilesional oedema and mass effect when compared to pretreatment images of Figure 2. However, the lesion was not yet resolved. **C** shows the MRS spectrum, which reveals recovery of the NAA peak, reduction of the Cho peak and not longer presence of lipids, when compared to pre-treatment spectra.

**Figure 5:**
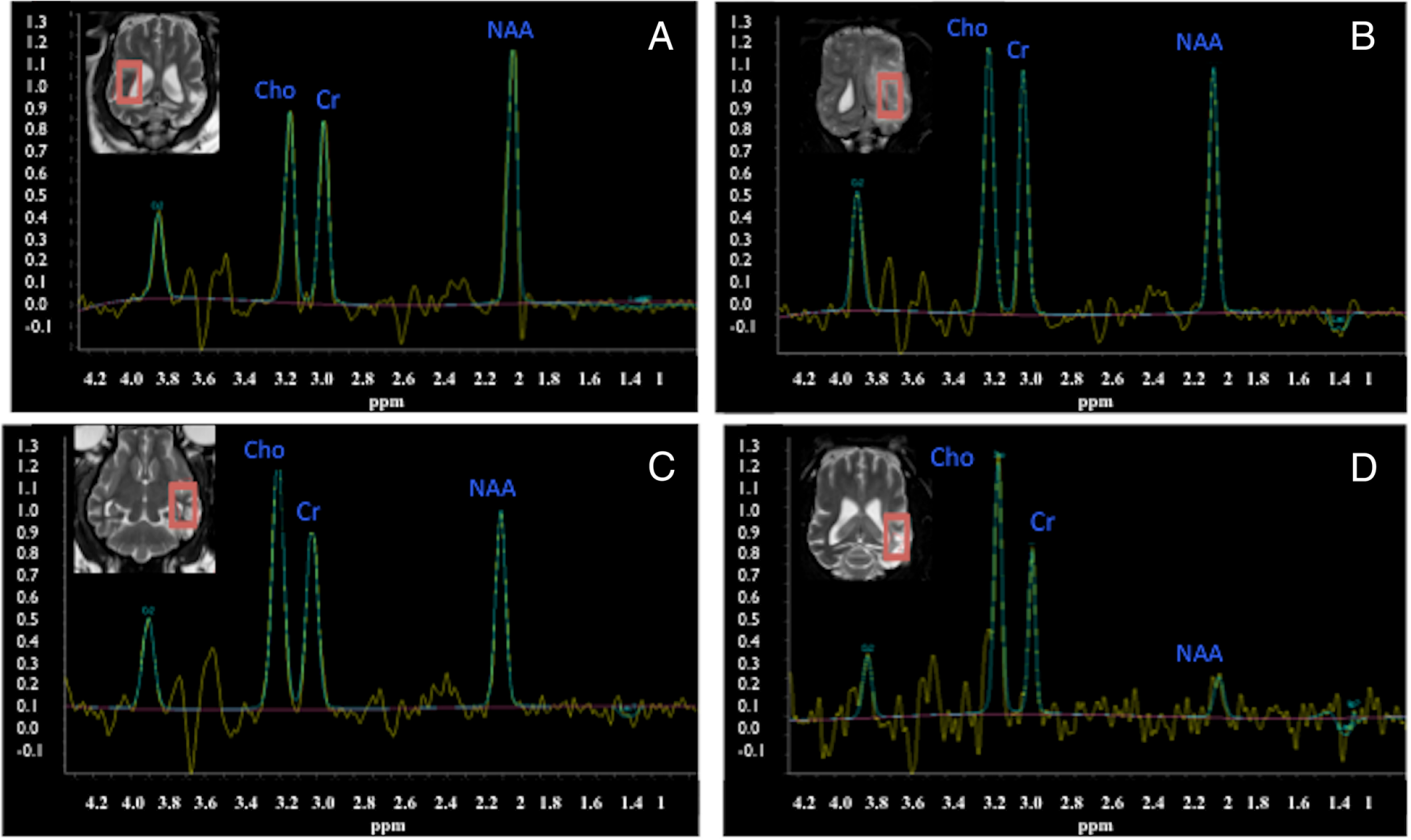
**H-1 MRS single voxel PRESS 144 in a Pug dog with multifocal MUO. A**: normal side before treatment, **B**: abnormal side before treatment, **C**: abnormal side straight after radiotherapy treatment, **D**: abnormal side 3 months after. Notice the decrease of the NAA concentration over time, which is very marked at 3 months.

All dogs included in this study had MRI follow-up immediately after the last session of RT. MRI showed marked reduction of the perilesional oedema in all cases. Mass effect was improved in 5 out of 6 dogs. One patient with a focal lesion in the brain stem showed complete resolution after RT (Figure 3). Two dogs with focal lesions showed a decrease in size of the lesion (Figure 4). In the dogs with multifocal disease, two had improvement and reduction in size of the lesions; while in one dog with multifocal disease the lesions were not improved (Figure 5). The signal intensity pattern of the remaining lesions in all dogs after treatment was the same as before treatment. No new abnormalities were observed in any of the patients included in this study.

H-1 MRS performed immediately after radiotherapy showed evidence of recovery of the NAA concentration in 5 cases (Figures 3 and 4). In these cases the choline concentration was either similar to pre-treatment or slightly reduced. One dog showed worsening on the metabolite concentration after treatment, especially reduction in the NAA concentration (Figure 5).

CSF in one dog was heavily contaminated with blood rendering proper analysis impossible. For this reason, it was not included in the results. CSF pleocytosis resolved in all other cases and protein content dropped to normal in 3 dogs and was reduced but above reference range in 2 dogs by the end of RT.

Except mild transient hair loss in the radiation field of one dog about 3 weeks after end of the therapy no side effects were observed during the follow-up period of more than 9 months. Prednisolone was reduced to 0.25-0.5 mg/kg once daily (SID) by the end of RT (12 days) and completely tapered in the next weeks (Table 2).

One dog was presented with relapsing neurological signs 4 weeks after the end of RT, the owner declined a further treatment and the dog was euthanased. Necropsy confirmed GME and a complete depletion of lymphocytes and lymphocytic cuffs at the site of the lesions was found. Co-irradiated regions in the brain, where no lesions were seen on MRI, had a normal histopathological appearance.

### Three-month follow-up

Five dogs were re-examined 3 months later. None of these dogs received steroids at that time point. The three dogs with the focal lesions on MRI and one of the dogs with multifocal lesions had a completely normal neurological examination and a NDS of 0. One of these dogs that initially presented due to seizures was free of seizures after therapy without concurrent antiepileptic treatment. The other dog with the multifocal lesions still showed unilateral menace response deficit and unilateral reduced proprioception, but was free of seizures during the 3 months of observation (NDS 1). This dog continued on antiepileptic medication (Phenobarbital 2.5 mg/kg BID, Levetiracetam 20 mg/kg three times daily (TID)) due to severe seizure activity before therapy. Serum levels of phenobarbital were checked 3 weeks after the beginning of the treatment and repeated every 3 months. Serum concentration was always within the therapeutic range.

MRI revealed improvement of the lesions (however, not complete resolution) in 4 out of 5 of the cases. No new lesions were found. MRS showed complete recovery of the Cho and NAA concentration, being equal to the contralateral side in 4 out of 5 dogs. In the dog without improvement of the MRI lesions, the NAA continued to decrease, while Cho and Cr concentrations remained stable during that time.

CSF analysis demonstrated a slight or moderate increase in total protein (0.33 g/l and 0.45 g/l, respectively) in 2/5 dogs.

### Long-term outcome

Five dogs had additional clinical follow-up examinations every 3 months for 9 (n = 1), 12 (n = 3) and 18 (n = 1) months after RT. Three dogs were neurologically normal in all follow-up examinations. One dog had one single seizure episode and one dog continued to have 2–3 seizures per month despite the use of antiepileptic medical treatment and the neurological deficits noticed at the 3 months follow-up were still present. This dog had a relapse 18 months after the end of RT, which was confirmed by MRI and CSF tap. The dog did not respond to prednisolone (4 mg/kg SID for 2 days, followed by 3 mg/kg) and cyclosporine (6 mg/kg BID) treatment and was euthanased 3 weeks later. The owner declined necropsy. The owners of the 4 other dogs were contacted by telephone: one dog was lost to follow-up after 12 months, the other 3 dogs were still alive and according to the owners remained clinically normal at the time of writing (12, 21 and 26 months after RT). Neither early nor late onset of RT reactions could be detected in our study population and owners described the whole RT plan as satisfactory and without any relevant complications.

In all patients we were always able to record good quality spectra from both the diseased and from the non-affected sites. All the acquired spectra were successfully studied further using the LC Model.

## Discussion

This pilot study shows that the 30 Gy ionizing RT combined with immunosuppressive dosage of corticosteroids is a feasible protocol that can be considered as a therapeutic option not only in dogs with focal but also in dogs with multifocal MUO. Furthermore we showed that H-1 MRS could be applied to strengthen the clinical suspicion achieved by means of combined data arising from clinical and neurological examination, CSF-centesis, conventional MRI and to monitor the disease course during the treatment of MUO.

The survival time in our study (median of 476 days) is in accordance with the findings by Munana *et al.* [31], who found that six out of seven dogs with GME treated with radiotherapy had a survival time of > 250 days with a median of 404 days. Munana *et al.* [31] included mainly dogs with focal GME. Only one dog with multifocal clinical signs was enrolled in that study and was treated with RT. It was euthanased immediately after the completion of RT due to a lack of clinical response. In our study, we included dogs with MRI features of focal and multifocal MUO. All our patients with focal MUO responded favourably to RT treatment similarly to those from the study of Munana *et al.* [31]. In contrast to the only study about RT in focal GME patients, we were able to follow patients clinically and radiologically up to 26 months. Similar to Munana *et al.* [31] all our dogs received steroids during the study period. But since neither the exact dosage nor the duration of treatment is described in their paper, we cannot compare the effect of this add-on treatment for both studies. The same holds true for most studies about medical treatment of MUO. In one study prednisolone monotherapy (11 dogs) was compared to prednisolone in combination with Lomustine (14 dogs) [34]: within the first 12 months of treatment, the dosage of prednisolone was decreased from 1.4 to 0.6 mg/kg/d compared to a dosage reduction from 2.1 to 0.2 mg/ kg/d in dogs treated with both. In only 4 dogs (all Lomustine group) could the prednisolone be discontinued in that study and in only one dog were the steroids discontinued after 3 months [35]. In one report about NME in 7 dogs treated either with prednisolone alone or in combination with cyclosporine [36], the 4 dogs receiving both medications prednisolone were started with 1 mg/kg twice daily (BID), reduced after two weeks to 0.75 mg/kg and after another 2 weeks reduced to the final dosage of 0.5 mg/kg BID. In the dogs with monotherapy steroid dosage was not reduced. In a more recent study [20] prednisolone was used in combination with cytosine arabinoside for treatment of MUO: the initial dosage of 1 mg/kg BID was completely tapered over 34 weeks. The dosage after 3 months was 0.25 mg/kg BID. In only one case series, prednisolone was completely replaced by cyclosporine within 2 weeks in dogs with suspected GME. In comparison to these results, the steroids in our dogs were all discontinued within 3 months after the beginning of RT.

The MRI findings in this study agree with previously reported findings [2,17-20]. Lowrie *et al*. [20] studied the prognostic factors and outcome of dogs with MUO. These authors showed that MRI resolution of lesions at three months are indicative of a good outcome, but not predictive of relapse, whilst abnormal CSF at three months was associated with higher risk of relapse. They concluded that the best prognostic factors were a combination of repeated MRI and CSF analysis, with resolution of both at three months suggesting a good or excellent long-term outcome. Only one dog in our study had complete MRI resolution of the lesions previously seen over 3 months. However, H-1 MRS showed in 5 cases that the NAA recovered over time while the choline concentration decreased and lipids were not present anymore. These findings parallel the improvement on the clinical status and the CSF analysis in four out of five dogs. Decreased levels of NAA are well documented in people with inflammatory lesions, such as multiple sclerosis [24,37]. The reduction of NAA in acute lesions may be caused by oedema, transient neuronal impairment or loss [24]. NAA can show a dynamic behaviour over time as it may recover to remyeliniation [24,37] or attributed to therapy in viral diseases [38]. In people a decrease of NAA correlates well with increasing neurological disability and worsening clinical state [39]. Choline is reported to be mildly to moderately elevated in acute demyelination due to alteration of the cell membranes and due to increased membrane turnover in viral diseases. These findings may recover to normality during the follow-up after acute events in multiple sclerosis [37], or may increase during the progress of viral disease [38]. The presence of lipids is attributed to membrane breakdown, when fractured lipids become spectroscopically visible. Lactate is related to anaerobic glycolysis of the brain or macrophage activation after membrane breakdown [13,15]. In this scenario, the changes seen in our patients corresponded to inflammatory white matter disease in people. Furthermore, the follow-up studies allowed us to demonstrate the recovery of NAA (and decrease of Cho in some cases), which can be correlated, with the improvement of the clinical signs and neurodisability score.

This is the first report of dogs with MUO in which H-1 MRS is used as a diagnostic and monitoring tool. Several authors have acknowledged the need of a non-invasive test for the diagnosis of MUO [2,20,34]. The preliminary results of this pilot study show that H-1 MRS is a feasible technique and provides additional information for the diagnosis of MUO and it is a valuable tool for monitoring the disease. Further studies with larger number of patients are necessary to assess its validity as a prognostic factor.

H-1 MRS is also used to evaluate the potential changes of normal brain tissue caused by radiotherapy [40-42]. At the three-month follow-up, we did not observe any change in the normal contralateral side in any dog. In this study no relevant side effects of RT could be detected during the observation period. The fractionation schedule of 30 Gy in 10 fractions is considered to be standard for human patients with whole brain radiation due to metastatic disease. With the used protocol of 30 Gy in 10 fractions the late toxicity such as radiation-induced dementia is estimated to be 1.9%-5.2% for human patients treated with brain metastasis, and is not high enough to warrant withholding quality of life prolonging whole brain RT [43].

A potential downside of this treatment protocol is the need for repeated general anesthesia for RT and CSF collection. However, this is an inevitable part of this treatment protocol and we were not aware of any detrimental effects in the present study. The risk of complications due to the daily short and mild anaesthesia can be considered low; no increased risks for dogs with intracranial neoplasia undergoing RT have been reported in the literature.

A number of limitations are associated with this pilot study. This study was not designed to assess the superiority of the 30 Gy RT protocol against another treatment. Moreover the low number of dogs included and the lack of a control group treated only with corticosteroids or with other drugs highlights the need for further evidence-based studies. Therefore, we cannot prove how much of the observed improvement in our patients was due to steroid treatment or was a result of the RT or a combination of both. To achieve this, a larger randomized, blinded, superiority study is needed to draw meaningful conclusions. Another limitation of the study is that the radiologist was not blinded to the clinical signs. However, the diagnosis of MUO should be a combination of the knowledge of the clinical signs, MRI and CSF findings. Histological confirmation before initiation of treatment might be advocated as the “*condicio sine qua non*” in order to design more informative studies about treatment of MUO. The possible complications [44], owners concerns, and technical difficulties (such as sampling vulnerable anatomical location or achieving a definitive diagnosis) are important drawbacks. Biopsy was strongly suggested to the owners of all our patients but none agreed to the procedure. Therefore we concur with Granger *et al*. [3] that the definition of inclusion criteria based upon clinical characteristics alone is important in order to include a high number of dogs in such studies.

## Conclusions

RT with a total dosage of 30 Gy in combination with corticosteroids is a feasible potential additional option for the treatment of MUO compared to previously published protocols. A randomized, prospective study, comparing outcome after RT and medical treatment, will be the next step to approve the clinical validity of RT. Furthermore, H-1 MRS is a promising non-invasive technique also in veterinary neuroradiology that provides very useful biochemistry information and might be used in the future as a tool for assessing prognostic factors and treatment effectiveness.

## Abbreviations

3DCRT: 3D conformal radiation therapy
BID: (bis in die) twice daily
Cho: Choline
Cr: Creatine
CSF: Cerebrospinal fluid
CT: Computertomography
CTV: Clinical target volume
ELISA: Enzyme linked immunosorbent assay
GME: Granulomaous meningoencephalitis
Gy: Gray
H-1 MRS: Proton magnetic resonance spectroscopy
IGRT: Image guided radiotherypay
IMRT: Intensity modulated treatment
MRI: Magnetic resonance imaging
MUO: Meningoencephalitis of unknown origin
NAA: N-acetyl aspartate (NAA)
NCC: Nucleated cell count
NDS: Neurodisability score
NLE: Necrotizing leukoencephalitis
NME: Necrotizingmeningoencephalitis
PCR: Polymerase chain reaction
PLR: Pupillar light reflex
PTV: Planning target volume
RT: Radiation therapy
SID: (*semel in die*) once daily
TID: (*ter in die*) three times daily
TP: Total protein
WBCs: White blood cells
